# Dilemmas in the reliable estimation of the *in-vitro *cell viability in magnetic nanoparticle engineering: which tests and what protocols?

**DOI:** 10.1186/1556-276X-7-77

**Published:** 2012-01-16

**Authors:** Clare Hoskins, Lijun Wang, Woei Ping Cheng, Alfred Cuschieri

**Affiliations:** 1 Institute for Medical Science and Technology (IMSaT), Wilson House, 1 Wurzburg Loan, University of Dundee, Dundee, DD2 1FD, UK; 2 School of Pharmacy, University of Hertfordshire, College Lane, Hatfield, Hertfordshire, AL10 9AB, UK

**Keywords:** magnetic nanoparticle, cellular interaction, cytotoxicity, cell viability assay, zeta potential.

## Abstract

Magnetic nanoparticles [MNPs] made from iron oxides have many applications in biomedicine. Full understanding of the interactions between MNPs and mammalian cells is a critical issue for their applications. In this study, MNPs were coated with poly(ethylenimine) [MNP-PEI] and poly(ethylene glycol) [MNP-PEI-PEG] to provide a subtle difference in their surface charge and their cytotoxicity which were analysed by three standard cell viability assays: 3-(4,5-dimethylthiazol-2-yl)-5-(3-carboxymethoxyphenyl)-2-(4-sulfophenyl)-2H-tetrazolium [MTS], CellTiter-Blue and CellTiter-Glo (Promega, Southampton, UK) in SH-SY5Y and RAW 264.7 cells The data were validated by traditional trypan blue exclusion. In comparison to trypan blue manual counting, the MTS and Titer-Blue assays appeared to have consistently overestimated the viability. The Titer-Glo also experienced a small overestimation. We hypothesise that interactions were occurring between the assay systems and the nanoparticles, resulting in incorrect cell viability evaluation. To further understand the cytotoxic effect of the nanoparticles on these cells, reactive oxygen species production, lipid peroxidation and cell membrane integrity were investigated. After pegylation, the MNP-PEI-PEG possessed a lower positive surface charge and exhibited much improved biocompatibility compared to MNP-PEI, as demonstrated not only by a higher cell viability, but also by a markedly reduced oxidative stress and cell membrane damage. These findings highlight the importance of assay selection and of dissection of different cellular responses in *in-vitro *characterisation of nanostructures.

## Background

Magnetic nanoparticles [MNPs] have assumed importance for the imaging of diseases such as cancer and diabetes [1]. In the field of tissue engineering, magnetic nanoparticles have been previously reported for many applications including cellular labelling, sorting and monitoring, targeted *in-vivo *therapeutic delivery, stem cell replacement therapy and welding tissue surfaces [2,3]. In 2007, Syková and Jenelová incorporated superparamagnetic nanoparticles into mesenchymal stem cells for the regeneration of tissue damage in the central nervous system [4]. They reported that the superparamagnetic nanoparticles enabled imaging and control of cellular migration by external magnetic fields to the wound site, optimised the number of cells needed and also helped monitor any possible side effects [4]. Some superparamagnetic nanoparticles were previously approved for imaging and therapeutic applications in humans, e.g. Feridex IV^® ^and Combidex^® ^(Advanced Magnetics Inc., Lexington, MA, USA) [5-7] and several other superparamagnetic nanoparticles are also undergoing phases I and II clinical trials [1,5,8,9].

Despite increased applications of MNPs emerging, little is known of the adverse biological side effects due to their nanoscale size. Recently, the importance of such biological characterisation and cytotoxic profile has been recognised. The use of appropriate assessments is vital in evaluating the biocompatibility of MNPs. At present, a range of assays are used which measure cell viability through non-specific enzyme activity (3-(4,5-dimethylthiazol-2-yl)-2,5-diphenyltetrazolium bromide [MTT], 3-(4,5-dimethylthiazol-2-yl)-5-(3-carboxymethoxyphenyl)-2-(4-sulfophenyl)-2H-tetrazolium [MTS], CellTiter-Blue assay) or via ATP level (CellTiter-Glo assay). However, little has been documented about the possible interactions between nanoparticles and the reagents used in these assays. The validation of these assays therefore merits urgent investigation.

Recently, Häfeli et al. reported a modification in their MTT assay used to measure the cytotoxicity of polyethylenoxide-coated magnetic nanoparticles [10]. Fisichella et al. reported that mesoporous silica nanoparticles interfered with the intracellular trafficking of the MTT formazan vesicles in HeLa cells and astrocytes, resulting in an overestimation of cytotoxicity when compared to flow cytometry [11]. In contrast, vast literature has been published on the effect of carbon nanotubes [CNTs] on such assays [12-18]. It has been documented that single-walled carbon nanotubes interfere with both absorbance and fluorescent cytotoxicity assays [12-14]. Belyanskaya et al. reported that the extent of interference can be attributed to three factors: (1) protocol of the assay, (2) surfactant coating and (3) chemical architecture of the CNT [12]. Belyanskaya reported that addition of one further centrifugation step at the end of the MTT assay in order to lyse the cells and discard the cellular components and CNT particles could reduce interferences caused by the CNTs. They concluded that extreme caution should be used when interpreting cell viability data without appropriate controls in place [12].

Here, we determine the suitability of various standard cell viability assays for a MNP with a particle size of 100 nm. As MNP surface properties especially surface charge is an important factor in determining their biocompatibility [19,20], the surface of commercial MNPs was modified with poly(ethylenimine) [MNP-PEI] and further conjugated with poly(ethylene glycol) [MNP-PEI-PEG]. PEI polymers have been reported to increase solution properties of nanoparticles and provide a platform for further modification, such as targeting or solubilisation moieties [21-23]. PEG has been widely accepted for nanostructure modification and drug delivery due to its biocompatibility and stealth properties *in vivo *[24-27]. Through surface modification, we obtained two types of MNPs with controlled difference on their surface charges, MNP-PEI having a higher positive charge than MNP-PEI-PEG, and used them as models to analyse the ability of various assays in discriminating the subtle difference in the level of effect those nanoparticles could impose to cells. Another consideration in assessing the sensitivity of the cell viability assays is MNP concentration. Concentrations of MNPs closer to physiological conditions and much lower than that reported in most studies were used in the present study. We validated the cell viability data obtained from commonly used MTS, CellTiter-Blue and CellTiter-Glo assays by comparing them with that obtained with traditional trypan blue exclusion to evaluate the suitability of those commercial cell viability assays for nanotoxicity studies. The validated data were further complemented by measurement of a number of cellular events in response to MNPs including cell membrane integrity, reactive oxygen species [ROS] production and lipid peroxidation [LPO] to give a more comprehensive safety profile.

## Methods

### Coating and characterisation of magnetic nanoparticles

MNPs (Chemicell GmbH, Berlin, Germany) diluted with 9.5 mL deionised water [DI] were sonicated for 18 h. *N*-(3-dimethylaminopropyl)-*N*-ethylcarboimide hydrochloride (5.7 mg) and *N*-hydroxysuccinimide (11.4 mg) dissolved in 1.9 mL of 2-(*N*-Morpholino)ethanesulfonic acid hemisodium salt [MES] buffer (0.5 M) were added to the solution and stirred for 1 h at room temperature [RT]. MES buffer (0.1 M) was added, and the solution was centrifuged at 40,000 × *g *at 4°C for 0.5 h. MNPs were resuspended in the MES buffer (0.1 M, 19 mL) containing 0.95 mg PEI (molecular weight [MW] 750,000) and stirred at RT for 3 h. Glycine (25 mM) in phosphate-buffered saline [PBS] (9.5 mL) was added, and the solution was stirred for a further 1 h at RT. The nanoparticles were 'washed' with DI (three times), and the resultant MNP-PEI eluted from the solution using a high-powered magnet. The nanoparticle pellet was resuspended in 10 mL of DI.

MNP-PEI (4 mL) was added to 0.08 M sodium tetraborate (12 mL) followed by methoxypolyethylene glycol *p*-nitrophenyl carbonate (MW 5,000) (20 mg) with stirring for 3 h at RT in the absence of light. The resultant solution was washed with DI, and the MNP-PLL-PEGs eluted from the solution using a high-powered magnet. The nanoparticles were resuspended in 4 mL DI.

Nanoparticle concentration was determined using inductively coupled plasma [ICP] analysis and dispersed in DI before sonication for 10 min before subsequent measurements. Hydrodynamic diameters, polydispersity index and zeta potential measurements were carried out using a photon correlation spectrometer (Zetasizer Nano-ZS, Malvern Instruments, Worcestershire, UK). All measurements were conducted in triplicate at 25°C, and an average value was determined. Prior to zeta potential analysis, standard control samples were run on the instrument.

### Culture of cell lines

Two cell lines, SH-SY5Y (human neuroblastoma; ATCC, Manassas, VA, USA) and RAW 264.7 (mouse macrophage), were used in our study. Neuroblastoma cells are being used to represent cells present at the regenerative site during nerve regeneration, and macrophage cells are the body's first line of defence for the immune system, phagocytosing foreign bodies and cleaning the blood of unknown particles; hence, any particles administered to the site of nerve injury will encounter these cells. SH-SY5Y cells were cultured in 50:50 Dulbecco's minimum essential medium [DMEM]: Ham's F-12 media containing 10% heat inactivated foetal bovine serum [FBS], 2 mM L-glutamine and 1% penicillin streptomycin [Penstrep] (all purchased from Invitrogen Ltd., Renfrew, UK). RAW264.7 cells (kindly donated by Prof. Colin Watts and Dr Alan Prescott, College of Life Sciences, University of Dundee, Dundee, UK) were cultured in DMEM containing 10% FBS, 2 mM L-glutamine and 1% Penstrep. Cells were grown under standard conditions (37°C and 5% CO_2_) to reach a confluency of 70% to 80% before being subjected to any further experimentation.

### Cellular uptake of nanoparticles measured by inductively coupled plasma

Cells seeded in 6-well plates and incubated with MNPs at final concentrations of 0, 1.56, 6.25 and 25 μg mL^-1 ^(the concentration of MNPs used for all experiments indicates the concentration of Fe^3+^) for 24 h. The medium was removed, and the cells were thoroughly washed with PBS for three times, trypsinised and resuspended in medium. The cell number was counted using a haemocytometer, and cells were placed in eppendorf tubes (1 × 10^6 ^cells/tube). The cell suspensions were centrifuged at 800 rpm for 5 min, and the supernatant was discarded. Concentrated hydrochloric acid (100 μL) was added to the cells, and the tubes were incubated at 90°C for 0.5 h. The samples were cooled to room temperature and centrifuged at 1,500 rpm for 10 min. The supernatant was diluted with deionised water and run on an ICP instrument (Optima 7000V DV, PerkinElmer, Wokingham, UK). A calibration was carried out using iron standard solutions of 0.5 to 5 μg mL^-1 ^(*R *= 0.9999). A control sample of deionised water was also run.

### Observation of cellular uptake of nanoparticles by transmission electron microscopy

MNPs were added to cells cultured in 75-cm^2 ^flasks (6.25 μg mL^-1^) for 24 h. The cells were washed with PBS (three times). A 10-mL fixative (4% paraformaldehdye, 2.5% gluteraldehyde in PIPES buffer, pH 7.2) was added to the flasks and incubated for 0.5 h at room temperature. The cells were scraped off the flask and centrifuged into a pellet. The pellet was set in resin, and micron-sized slices were cut. The specimens were viewed using transmission electron microscopy [TEM] (JEM.1200ex, JEOL Ltd., Herts, England, UK), and images were recorded in digital imaging plates and scanned in a Ditabis Micron scanner (Ditabis AG, Pforzheim, Germany).

### Cell viability determination by MTS, CellTiter-Blue and CellTiter-Glo assays

MTS and CellTiter-Blue are colorimetric and fluorescent assays (respectively) used to measure cell viability via non-specific redox enzyme activity. CellTiter-Glo is a luminescent assay used to measure cell viability by ATP level. Cells (100 μL, 1 × 10^5 ^cells/ml) were seeded into a 96-well flat-bottomed plate (white for Titer-Glo) and incubated for 24 h at 37°C with 5% CO_2_. The medium was replaced with increasing MNP concentrations (1.56 to 25 μg mL^-1^) (in triplicates). The cells were incubated for 24, 72, 120 and 168 h (for 120- and 168-hr incubations, the medium was replaced at 72 h with fresh medium containing appropriate concentrations of MNPs). The cells were washed (with PBS, three times) and replaced with fresh medium (100 μL). MTS (20 μL) or CellTiter-Blue (20 μL) reagents were added to the wells, and the plate was incubated for 4 h protected from light. Absorbance (MTS) was recorded at 490 nm, and fluorescence intensity (CellTiter-Blue) was recorded (excitation 560 nm, emission 590 nm). To eliminate possible interference between MNPs and assay readings, cells treated with same concentrations of MNPs but without addition of assay reagents were used as blank wells. Both assays were measured on a Tecan M200 multimode plate reader (Tecan Austria GmbH, Grödig, Austria). CellTiter-Glo reagent was added to the wells (50 μL and 50-μL media) and incubated at room temperature for 10 min protected from light. The luminescence was recorded using the same multimode plate reader. As per MTS and Titer-Blue assays, blank wells (with no reagents) were measured for luminescence and deducted from the values in experimental wells. Values of viability of treated cells were expressed as a percentage of that from corresponding control cells. All experiments were repeated at least three times. All assay kits were purchased from Promega, Southampton, UK.

### Trypan blue exclusion assay

Cells were seeded into a 12-well plate and incubated for 24 h at 37°C with 5% CO_2_. The cells were treated as previously described in MTS, Titer-Blue and Titer-Glo. The cells were washed with PBS three times and trypsinised. Trypan blue was added to a 100-μL cell suspension in an equal volume and incubated at room temperature for 5 min. The viable cells were counted using a Countess™ automated cell counter (Invitrogen, Ltd., Renfrew, UK). Values of viability of treated cells were expressed as a percentage of that from corresponding control cells. All experiments were repeated at least three times.

### Reactive oxygen species assay

Cells were seeded into a 96-well plate (10,000/well) and incubated for 24 h. Cells were incubated with increasing MNP concentrations (1.56 to 25 μg mL^-1^) for 1, 4, 24 and 72 h. The cells were washed three times with PBS and incubated for 1 h with 100-μM carboxy-H_2_DCFDA (Invitrogen Ltd., Renfrew, UK) in PBS at 37°C protected from light. The cells were washed three times with PBS and incubated with a 100-μL serum-free medium for a further 0.5 h. The medium was replaced with PBS. The fluorescence intensity of the samples was measured at 560 nm (excitation) and 590 nm (emission) on a Tecan M200 microplate reader (Tecan Austria). The percentage of dichlorofluorescin [DCF] fluorescence was calculated in respect to control cells assumed to be 100%.^a^

### Lipid peroxidation measurement by thiobarbituric acid reactive substance assay

Cells in an exponential growth phase were seeded into a 6-well plate and incubated for 24 h. The medium was replaced with increasing MNP concentrations (1.56 to 25 μg mL^-1^). After incubation, the medium was removed, and the wells were washed three times with PBS. Cells were trypsinised and resuspended in 0.5 mL PBS containing 0.05% butylated hydroxytoluene on ice. The cell suspensions were sonicated for 5 s three times at 40 V and were kept on ice. Malondialdehyde bis(dimethyl acetal) [MDA] standard solutions (0 to 5 μM) were prepared, and 100 μL of samples or standards was added to the Eppendorf tubes. Sodium dodecyl sulphate (100 μL, 2%) was added, and the tubes were incubated for 5 min at room temperature. Thiobarbituric acid (250 μL) was added to the eppendorf tubes before incubation at 95°C for 1 h. The samples were cooled on ice for 10 min and centrifuged at 3,000 rpm for 15 min at 4°C. The supernatant was pipetted into the wells of a 96-well plate, and fluorescent measurements were taken at 530 nm (excitation) and 550 nm (emission). The results were calculated as nanomoles of MDA per milligram of cellular protein. Protein content was determined by the addition of a 100-μL sample to a 3-mL Bradford reagent. The samples were mixed well at room temperature for 5 min, and absorbance was measured at 595 nm. The absorbance values were compared to a calibration curve carried out using bovine serum albumin, and the protein concentration was determined.^a^

### Cell membrane integrity analysis

Cells were seeded into a 96-well plate (15,000/well) and grown for 24 h. The medium was replaced with increasing magnetic nanoparticle concentrations (1.56 to 25 μg mL^-1^). The plates were incubated for 1, 4, 24 and 48 h. After incubation 2 μL of lysis buffer was added to the positive control wells, and the plate was centrifuged at 1,500 rpm for 10 min at 37°C. After centrifugation, 50 μL of the supernatant was removed from each well and placed into a new plate, and 50 μL of a membrane integrity assay reagent was added to the wells. The plates were incubated for 10 min at 37°C protected from light. Twenty-five microlitres of stop reagent was then added to the wells, and the fluorescence of the samples was measured at 560 nm (excitation) and 590 nm (emission) on the microplate reader. The percentage of cytotoxicity with respect to the positive control wells was calculated, whereby the lysed cells were assumed to have 100% lactate dehyrogenase [LDH] release.^a^

## Results

### Coating and characterization of magnetic nanoparticles

The particles were successfully coated with PEI and further functionalised with PEG. ICP was used to deduce the concentration of the MNPs after each reaction (based on the total iron content). The concentration after the initial coating with PEI was 1.39 mg mL^-1 ^(74% yield) and 1 mg mL^-1 ^(72% yield) after pegylation based on ICP analysis. After coating, the particles were noticeably more stable in the solution, especially after pegylation. The size of the MNPs was determined using photon correlation spectroscopy which measures the hydrodynamic radius of the particles in the solution [see Table S1 in Additional file 1]. The particles were measured at 0.25 mg mL^-1 ^in deionised water. With polymer coating, the size of the commercial Chemicell nanoparticles increased from 101 nm to 146 nm (MNP-PEI); the size further increased upon pegylation to 361 nm.

The zeta potential of the 'naked' MNPs was negative (-38.2 mV), due to the -COOH groups on the surface of the particles [see Table S1 in Additional file 1]. Addition of a cationic polymer such as PEI increases the overall zeta potential, resulting in a positive value. This assumption can be made due to the positive charge on the amine groups of the polymer backbone, binding to the -COOHs thus rendering them neutral, plus and unbound amine groups which will still hold their positive charge. The PEI coating gave a positive value (+17.7 mV) which indicated that the polymer had been successfully coated with the polymer, which is in agreement with the size data [see Table S1 in Additional file 1]. The zeta potential measurement for the pegylated particle was +12.1 mV. This value indicated that pegylation had occurred as the zeta potential increased in negativity compared to that of the MNP-PEI due to the presence of -OH groups on the particle surface which is due to the PEG coating (MNP-PEI-PEG) [see Table S1 in Additional file 1].

### Cellular uptake of nanoparticles

Table S2 in Additional file 1 shows the intracellular content of MNPs in SH-SY5Y and RAW 264.7 cells after 24 h incubation at different concentrations. In the SH-SY5Y cells, the cellular uptake was increased ninefold from 2.867 pg to 26.763 pg per cell (at 25 μg mL^-1^) upon PEI coating of the nanoparticles. After pegylation, an eightfold increase in cellular uptake was observed when compared to the uncoated MNPs at the same concentration. The increase in cellular uptake, which is dependent on surface properties of MNPs, further confirms the coating of the particles. Only a small increase in nanoparticle uptake was observed in RAW 264.7 upon coating (maximum of 1.6-fold and 1.7-fold increase by PEI and PEG, respectively) [see Table S2 in Additional file 1], thus indicating that the cellular uptake in RAW 264.7 cells is less affected by the surface properties of the nanoparticles compared to that in SH-SY5Y cells. This is consistent with the phagocytic nature of the RAW 264.7 cells. Compared to the SH-SY5Y cells, the RAW 264.7 cells achieved a lower cellular uptake; we postulate that the smaller cellular volume of the RAW 264.7 cells was a limiting factor compared with the much larger volume of SH-SY5Y cells.

**Figure 1:**
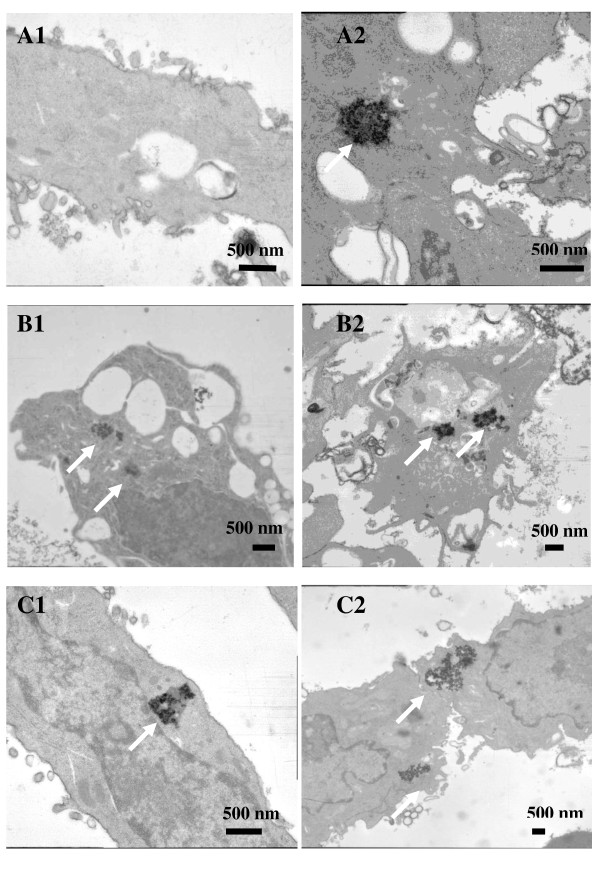
**TEM images show cellular uptake of magnetic nanoparticles**. *A1 *MNP incubated with SH-SY5Y cells, *A2 *MNP incubated with RAW 264.7 cells, *B1 *MNP-PEI incubated with SH-SY5Y cells, *B2 *MNP-PEI incubated with RAW 264.7 cells, *C1 *MNP-PEI-PEG incubated with SH-SY5Y cells and *C2 *MNP-PEI-PEG incubated with RAW 264.7 cells. Samples were incubated with cells at 6.25 μg mL^-1 ^for 24 h, and internalisation of nanoparticles was analysed by TEM as described in the 'Methods' section.

The TEM micrographs were consistent with the ICP data, for the SH-SY5Y cells increased numbers of intracellular nanoparticles were evident upon coating the particles with PEI and PEG (Figure 1A1, B1, C1). The RAW 264.7 images (Figure 1A2, B2, C2) were also in good agreement with the ICP measurement whereby PEI and PEG coating had less effect on the total cellular uptake of the nanoparticles.

### Cell viability measured by MTS, CellTiter-Blue and CellTiter-Glo assays

**Figure 2:**
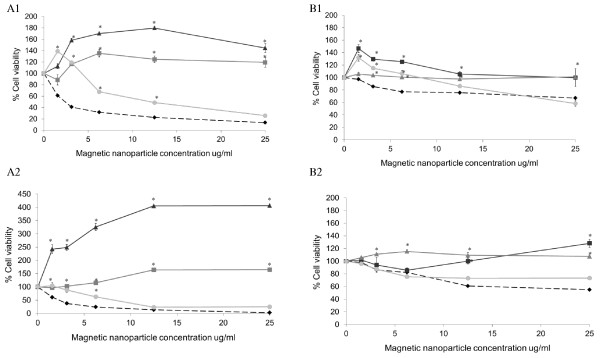
**Cell viability of SH-SY5Y and RAW 264.7 cells**. *A1 *SH-SY5Y cells incubated with MNP-PEI nanoparticles, *A2 *RAW 264.7 cells incubated with MNP-PEI nanoparticles, *B1 *SH-SY5Y cells incubated with MNP-PEI-PEG nanoparticles and *B2 *RAW 264.7 cells incubated with MNP-PEI-PEG nanoparticles. The cells were incubated at different concentrations as indicated over a 72-h incubation. Cell viability was determined using common assays including MTS assay (square), CellTiter-Blue assay (triangle), CellTiter-Glo assay (circle) and trypan blue counting (diamond). (*n *= 3 ± SE). Asterisk denotes significantly increased level of cell viability compared with trypan blue measurement (*p *< 0.05).

An interesting phenomenon was observed when measuring the cell viability of both the SH-SY5Y (Figure 2A1, B1) and RAW 264.7 (Figure 2A2, B2) cells after incubation with the MNPs using MTS, CellTiter-Blue and CellTiter-Glo assays. At increased concentrations, the MNPs became attached to either the cell membrane or the bottom of the plate, appearing as a brown colour in the well (even after five washes with fresh culture media) (data not shown). The presence of these MNPs resulted in greater absorption readings in the MTS assay and thus significantly showed an overestimation of the cell viability (*p *< 0.05). This phenomenon could be explained by the adherence of the sticky polymers to the well surface or the positive amine groups being attracted to the negative charge of the cell membrane. After pegylation, the interference appeared to have been reduced; however, the value of cell viability that appeared was still larger compared to the visual inspection of viable cells under microscope (data not shown).

In CellTiter-Blue assay, viable cells reduce resazurin into fluorescent resorufin. Similar to the MTS assay, the polymer-coated MNPs caused a significant increase in the fluorescent measurement (*p *< 0.05 with exception to 1.56 μg mL^-1 ^MNP-PEI-PEG on SH-SY5Y cells), giving an overexpression with a similar trend in time dependency (Figure 2). This indicated that the presence of the nanoparticle in cellular environments increased the fluorescent intensity exhibited by the resorufin dye.

The results for the CellTiter-Glo assay (Figure 2) showed a similar trend to the MTS and CellTiter-Blue assays. Overall, with increased time and concentration, no significant difference from the actual cell viability was observed (based on trypan blue exclusion, see below) (*p *> 0.05). This was observed for both cell lines.

This unique phenomenon could be due to a number of factors, either the presence of the MNPs both intracellular and on the membrane of the cells or the nanoparticles themselves interfere with the reagents. In our experiments, wells with nanoparticles (intracellular or on the cell membrane) and without assay reagents were used as blanks and were deducted from experimental wells. Furthermore, nanoparticles with assay reagents in the absence of cells were also analysed, and no significant effect on absorbance, fluorescence or luminescent readout was observed (data not shown). Therefore, we hypothesise that the increased absorbance and fluorescence (and to a lesser extent, luminescence) were only evident and elicited by the combination of cells, nanoparticles and assay reagents.

### Validation of magnetic nanoparticle cytotoxicity with trypan blue exclusion

**Figure 3:**
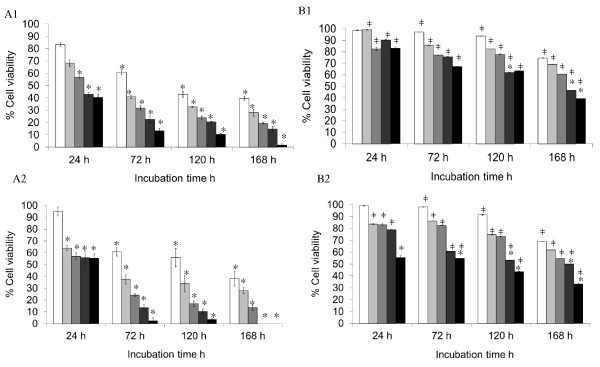
**Cell viability assessed by trypan blue exclusion**. Nanoparticles MNP-PEI and MNP-PEI-PEG at 1.56 (white bar), 3.125 (light grey bar), 6.25 (grey bar), 12.5 (dark grey bar) and 25 μg mL^-1 ^(black bar) were incubated with SH-SY5Y and RAW 264.7 cells over a period of 168 h. *A1 *MNP-PEI nanoparticles incubated with SH-SY5Y cells, *A2 *MNP-PEI nanoparticles incubated with RAW 264.7 cells, *B1 *MNP-PEI-PEG incubated with SH-SY5Y cells and *B2 *MNP-PEI-PEG incubated with RAW 264.7 cells. Experiments were performed three times, and data were expressed as mean ± standard errors. Asterisk denotes significant decrease in viability compared with control cells; square denotes significant increase in cell viability compared to MNP-PEI samples at a similar concentration and incubation time (*p *< 0.05).

Based on the above observations, trypan blue exclusion was used as the gold standard method to validate the cell viability data obtained by the above assays (Figure 3). This method involved direct counting of viable cells and hence eliminated the possibility of interference from occurring. The results demonstrated a large difference between the cell viability data from trypan blue counting and all other three enzyme activity-based assays, especially those measured by MTS and CellTiter-Blue which involve a group of cellular redox enzymes (Figure 2). These values correlated well with visual estimations when observed under microscope. The trypan blue results clearly showed that the MNP-PEI-PEG possessed a significantly less cytotoxic effect to the cells compared with the MNP-PEI, highlighting the significance of surface charge of the nanoparticles in determining their biocompatibility (Figure 3).

### Cellular reactive oxygen species production and lipid peroxidation upon magnetic nanoparticle treatment

**Figure 4:**
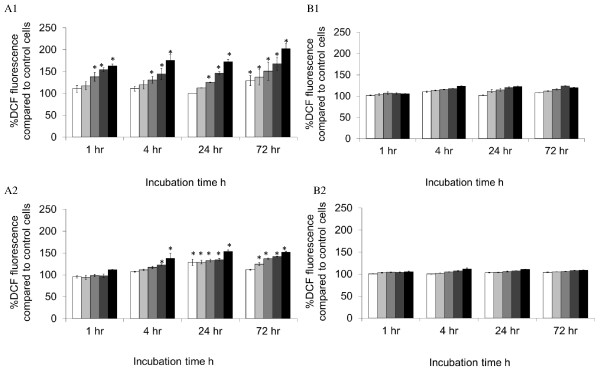
**ROS production in SH-SY5Y and RAW 246.7 cells**. The cells were incubated with MNP-PEI and MNP-PEI-PEG at 1.56 (white bar), 3.125 (light grey bar), 6.25 (grey bar), 12.5 (dark grey bar) and 25 μg mL^-^1 (black bar) over a period of 72 h. *A1 *SH-SY5Y cells incubated with MNP-PEI, *A2 *RAW 246.7 cells incubated with MNP-PEI, *B1 *SH-SY5Y cells incubated with MNP-PEI-PEG, *B2 *RAW 246.7 cells incubated with MNP-PEI-PEG. Data were expressed as a percentage of control cells. (*n *= 3 ± SE). Asterisk denotes significant increase in the percentage of DCF fluorescence compared with control cells (*p*, 0.05).

The above data indicated that commercially available, standard cell viability assay kits may not be suitable for most nanotoxicity studies. To assess other cellular events that could contribute to the evaluation of nanotoxicity, we analysed the oxidative stress induced by MNPs. All results were expressed as a percentage of control cells which were assumed to be 100%. MNP-PEI induced ROS production in a dose-dependent manner (Figure 4). The ROS level was increased to twofold by MNP-PEI (25 μg mL^-1^) in SH-SY5Y cells after 72 h compared to that in the control cells. The maximum induction of ROS in RAW 264.7 cells was, however, lower than that in SH-SY5Y cells (50% increase on control) (25 μg mL^-1^, 24 h), probably reflecting the relatively higher basal level of intracellular free radicals in macrophages [28]. Strikingly, pegylated nanoparticles did not appear to affect the ROS level in both the SH-SY5Y and RAW 264.7 cells at lower concentrations. At 25 μg mL^-1^, a small increase of ROS by MNPs in SH-SY5Y cells was observed compared to that in the control cells at a 4-h exposure point and remained consistent thereafter.

**Figure 5:**
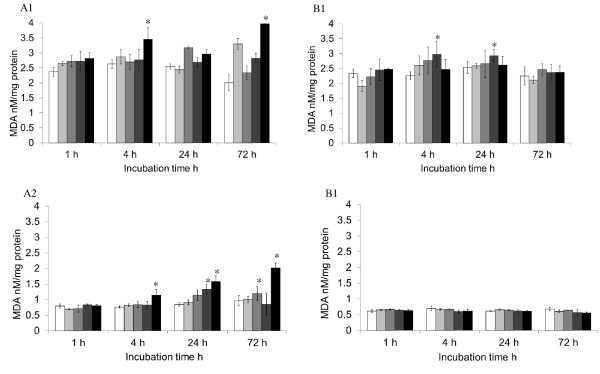
**Lipid peroxidation of SH-SY5Y and RAW 246.7 cells**. LPO of the cells in response to MNP-PEI and MNP-PEI-PEG at 1.56 (white bar), 3.125 (light grey bar), 6.25 (grey bar), 12.5 (dark grey bar) and 25 μg mL^-1 ^(black bar) over a period of 72 h. *A1 *LPO of SH-SY5Y cells in response to MNP-PEI, *A2 *LPO of RAW 246.7 cells in response to MNP-PEI, *B1 *LPO of SH-SY5Y cells in response to MNP-PEI-PEG and *B2 *LPO of RAW 246.7 cells in response to MNP-PEI-PEG. LPO values were calculated and expressed as nanomoles of MDA per milligram of cellular protein. (*n *= 3 ± SE.). Asterisk denotes significant increase compared to control cells (*p*, 0.05).

One of the major damage by the elevated level of ROS in cells is oxidation of polyunsatured fatty acids in lipid (lipid peroxidation, [LPO]). The results from the thiobarbituric acid reactive substance assay (Figure 5) showed a similar trend in the level of LPO to that of the ROS in response to MNP treatment (Figure 4). In general, at higher concentrations and longer incubation times, both the SH-SY5Y and RAW 246.7 cells produced increased levels of LPO when treated with MNP-PEI. After pegylation, the nanoparticle-induced membrane stress or degradation was again greatly reduced. These results suggested that the primary amines on the PEI backbone which attribute to the positive charge on the MNP surface play an important role in inducing cellular oxidative stress. The data also indicated that pegylation of nanoparticles improves their intracellular stability [29], and hence, with comparable cellular iron content [see Table S2 in Additional file 1], less free iron is released to the cytosol in MNP-PEI-PEG-treated cells so that cellular oxidative stress was reduced [30].

### Effect of magnetic nanoparticles on cell membrane integrity

**Figure 6:**
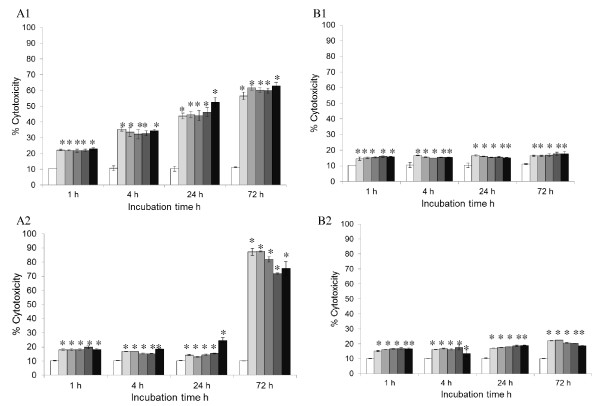
**Cell membrane integrity analysis via LDH leakage from cells**. Assay carried out in SH-SY5Y and RAW 264.7 cells incubated with MNP-PEI and MNP-PEI-PEG at 0 (white bar), 1.56 (light grey bar), 3.125 (grey bar), 6.25 (dark grey bar), 12.5 (very dark grey bar) and 25 μg mL^-1 ^(black bar) over a period of 72 h (*n *= 3 ± SE). *A1 *SH-SY5 cells incubated with MNP-PEI, *A2 *RAW 264.7 cells incubated with MNP-PEI, *B1 *SH-SY5 cells incubated MNP-PEI-PEG and *B2 *RAW 264.7 cells incubated with MNP-PEI-PEG. Results were calculated and compared to the positive control, whereby cells were lysed and 100% LDH release was assumed. Asterisk denotes significant increase compared with basal levels of LDH (*p*, 0.05).

Elevated levels of ROS and LPO could cause damage to the biological membrane. The membrane integrity assay measures the amount of LDH leakage from the cell into the culture media. Figure 6A1, A2 suggests that after 1 h incubation with MNP-PEI, 5% to 10% of the cell membrane had already experienced disruption in both SH-SY5Y and RAW 264.7 cells, when taking into account that the basal level of LDH in culture media was about 10% of the control ('total' LDH released to the media). The LDH leakage in SH-SY5Y cells increased with the incubation time of the MNP-PEI to a maximum of 50% after 72 h; however, no concentration dependency was exhibited at each time point (Figure 6A1). The cytotoxic effect of MNP-PEI on the RAW 264.7 cells remained mostly below 10% at 1, 4 and 24 h; however, a large increase in LDH leakage was observed at 72 h where approximately 70% cell membrane damage effect was observed (sevenfold increase from the basal level). Again, the membrane disruption appeared to be independent of nanoparticle concentration (Figure 6A2).

When both the SH-SY5H and RAW 264.7 cells were incubated with the MNP-PEI-PEG nanoparticles (Figure 6B1, B2), a small but constant (and significant *p *> 0.05) membrane disruption was evident. The amount of LDH leakage did not appear to be concentrated or time-dependent. The cytotoxic effect was consistently less than 10%, indicating that the pegylation of the nanoparticles greatly reduced their ability to damage the cell membrane.

## Discussion

In this study, we successfully coated MNPs with PEI and further modified them with PEG. The zeta potential measurements for surface charge correlated well with the polymer-coupled nanoparticles [see Table S1 in Additional file 1]. Cellular uptake results [see Table S2 in Additional file 1] for both the SH-SY5Y and RAW 264.7 cells further confirmed the polymer attachment as the particles coated with the PEI and PEI-PEG had more favourable surface properties and resulted in a similar increase in cellular uptake compared to the uncoated nanoparticles.

The cytotoxicity of the polymer-coated nanoparticles was determined using three commonly used cytotoxicity assays: MTS, CellTiter-Blue and Cell-Titer-Glo (Figure 2). Our findings suggest that none of these three assays were suitable for measuring the cytotoxicity of the nanoparticles studied. In contrast to Häfeli's findings [10], MTS and Titer-Blue assays gave large overestimations of the cell viability in both SH-SY5Y and RAW 264.7 cells when compared to trypan blue exclusion. However, the Titer-Glo assay appeared to give the closest readings to those obtained with trypan blue exclusion (Figure 2). It is important to note that a direct comparison is not appropriate between these assays as they are testing enzyme activities in different cellular entities; however, from these results, general observations have been made.

In our experience, this phenomenon is neither unique to PEI nor to the MNPs as we obtained similar observations with other polymers (poly(L-lysine), chitosan, PEG) with homemade nanoparticles (data not shown). As apparent in our studies, the interference was reduced, but not eliminated with pegylation of the cationic polymer-coated particle. In an effort to overcome this interference, we coated wells with 0.2% *w*/*v *silica solution to prevent the polymer-coated nanoparticles from attaching to the plates [31]. The results showed that using the 0.2% *w*/*v *silica solution did decrease the adhesive effect of the nanoparticles on the well surface; however, all three assays still overestimated the cell viability (data not shown). We hypothesised that if the MNPs were sticking to the cell membrane, then lysing cells after incubation with MTS reagents followed by centrifugation (removing cellular debris) could eliminate the interference in reading from the nanostructure. With these additional steps, the interference was reduced, but not eliminated (data not shown). We propose that for MTS and CellTiter-Blue assays, the false increase in viability might be due not only from the physical interference by the nanoparticles (not supported by our data) but also from changes in cellular activities involved in redox reactions in response to MNPs. This hypothesis merits further study.

There are many unknown factors that may influence the determination of the cytotoxicity profile of nanostructures [32]. Recently, the EU NanoSafety Cluster group has suggested that at least four methods of determining cytotoxicity should be used in order to obtain a reliable safety profile for novel nanomaterials. The present study has suggested that for our nanoparticles, using trypan blue exclusion is the only accurate method for determining cell viability (Figure 3). Other methods which are not dependent on cellular redox activities such as [^3^H]-thymidine incorporation and flow cytometry should be also considered when studying nanotoxicity. We have shown a reduction in toxicity after pegylation of the MNPs, which could be due to a number of factors including an increase in the coating diameter upon addition of the PEG moiety [33], thus increasing the stability of the nanoparticles and further shielding the cells from the iron oxide core. The cationic charge on the PEI is decreased upon pegylation due to a reduction in the primary amines on the polymer backbone hence decreasing the cytotoxic effect on the cells [34]. Finally, the presence of the PEG moiety may provide 'stealth' properties as has been widely reported [35-38].

In order to achieve a more in-depth understanding of the degree of cytotoxicity of the MNPs, we also investigated the effect the polymer-coated nanoparticles had on the integrity of the cell membrane and oxidative stress the MNPs elicited in cells. The ROS level increased significantly (*p *< 0.05) in both SH-SY5Y and RAW264.7 cells when exposed to MNP-PEI, overall in a dose-dependent manner (Figure 4). LPO in cells exhibited a similar pattern but slightly more fluctuating in SH-SY5Y cells (Figure 5). These increased ROS and LPO levels were not apparent upon incubation with MNP-PEI-PEG. The LDH leaking data showed membrane damage which was independent of nanoparticle concentrations for MNP-PEI. This is contradictory to the cell viability (Figure 3) of MNPs which was clearly dose- and time-dependent. The trypan blue and LDH assays work on the same principal, porous membranes allowing the passing of molecules. However, the size cutoff for trypan blue uptake and LDH leakage is not known, and this could account for the difference in data. We propose that a combination of oxidative stress, membrane disruption and possibly other factors contributed to the decrease of cell viability by MNP-PEI. In contrast, for MNP-PEI-PEG, the loss of cell viability over this time period could be attributed to cellular events other than oxidative stress and cell membrane damage and requires further study. As both SH-SY5Y and RAW264.7 cells possess complex functions due to their origin, other factors in both intra- and extracellular environments could be involved in the cellular responses. More studies to investigate these factors are underway in our laboratory.

## Conclusion

Our findings show that great caution should be exercised when interpreting cell viability data from common commercial assays on novel nanoparticulates. Our data strongly suggested that nanotoxicity analysis requires a different approach compared to conventional toxicity studies used for cytotoxic drugs and other molecules which are usually much less complex and smaller than nanostructures. Our results also indicate that cell viability measurement should be complemented with analysis of other cellular events when interpreting the cytotoxicity and biocompatibility profile of nanoparticles.

## Competing interests

The authors declare that they have no competing interests.

## Authors' contributions

CH carried out the coating, characterisation and cell experiments and wrote the paper. LY supervised the work. WPC carried out the photon correlation spectroscopy and zeta potential measurements, and AC was a scientific advisor. All authors read and approved the final manuscript.

## Endnotes

^a^ROS, LPO and LDH assays were previously checked for interferences and found to be unaffected by the nanoparticle presence. All statistical significance was assessed using *t *test analysis via Microsoft^® ^Excel^® ^2010 (Microsoft Corporation, Redmond, WA, USA) to a 95% confidence interval.

## Supplementary Material

Additional file 1 **Supplementary tables**. Two tables showing the physiochemical properties of MNP and polymer-coated MNP carried out by ICP analysis and photon correlation spectroscopy and the cellular uptake of MNP and polymer-coated MNP in SH-SY5Y and RAW 264.7 cells at 0, 1.56, 6.25, 25 μg mL^-1 ^over 24 h (*n *= 3). Click here for file
