# Supplementary material for: Dilemmas in the reliable estimation of the in-vitro cell viability in magnetic nanoparticle engineering: which tests and what protocols?
Source: Nanoscale Res Lett. 2012 Jan 16;7(1):77. doi: 10.1186/1556-276X-7-77 (PMC3275459; doi:10.1186/1556-276X-7-77)
Supplement: Additional file 1 — Supplementary tables. Two tables showing the physiochemical properties of MNP and polymer-coated MNP carried out by ICP analysis and photon correlation spectroscopy and the cellular uptake of MNP and polymer-coated MNP in SH-SY5Y and RAW 264.7 cells at 0, 1.56, 6.25, 25 μg mL-1 over 24 h (n = 3). [file 1556-276X-7-77-S1.DOC]

**Table 1.** Physiochemical properties of MNP and polymer coated MNP carried out by ICP analysis and photon correlation spectroscopy.

| Particle | Theoretical Concentration (mgmL^-1^) | Actual Concentration (mgmL^-1^) | Hydrodynamic Radius (nm) | PDI | Zetapotential | SD |
| --- | --- | --- | --- | --- | --- | --- |
| MNP | - | 18.78 | 101.3 | 0.056 | -38.2 | 0.544 |
| MNP-PEI | 1.88 | 1.39 | 145.8 | 0.164 | +17.7 | 5.98 |
| MNP-PEI-PEG | 1.39 | 1.00 | 361.4 | 0.248 | +12.1 | 1.67 |

**Table 2.** Cellular uptake of MNP and polymer coated MNP in SH-SY5Y and RAW 264.7 cells at 0, 1.56, 6.25, 25 μgmL^-1^ over 24 h (n=3). Concentration determined by ICP analysis (calibration R^2^= 0.997).

| Particle | Concentration of nanoparticles added, ugmL^-1^ | SH-SY5Y | | RAW 264.7 | |
| --- | --- | --- | --- | --- | --- |
|  |  | Concentration of Fe^3+^ uptake per cell, pg (± SE) | Increment increase from MNP | Concentration of Fe^3+^ uptake per cell, pg (± SE) | Increment increase from MNP |
| MNP | 0.00 | 0.560 (0.017) | - | 0.677 (0.084) | - |
|  | 1.56 | 0.503 (0.079) | - | 1.020 (0.007) | - |
|  | 6.25 | 1.070 (0.039) | - | 1.590 (0.014) | - |
|  | 25.00 | 2.867 (0.084) | - | 6.417 (0.026) | - |
| MNP-PEI | 0.00 | 0.560 (0.017) | - | 0.677 (0.084) | - |
|  | 1.56 | 2.663 (0.043) | 5-Fold | 1.127 (0.032) | 1.1-Fold |
|  | 6.25 | 10.783 (0.683) | 10-Fold | 1.767 (0.127) | 1.1-Fold |
|  | 25.00 | 26.763 (0.488) | 9-Fold | 10.507 (0.992) | 1.6-Fold |
| MNP-PEI-PEG | 0.00 | 0.560 (0.017) | - | 0.677 (0.084) | - |
|  | 1.56 | 1.767 (0.039) | 3-Fold | 0.897 (0.013) | 0.8-Fold * |
|  | 6.25 | 5.737 (0.039) | 5-Fold | 2.057 (0.012) | 1.3-Fold |
|  | 25.00 | 23.407 (1.092) | 8-Fold | 10.673 (1.269) | 1.7-Fold |

* Decrease compared to naked MNP.
